# Is Harbor Porpoise (*Phocoena phocoena*) Exhaled Breath Sampling Suitable for Hormonal Assessments?

**DOI:** 10.3390/ani11030907

**Published:** 2021-03-22

**Authors:** Anja Reckendorf, Marion Schmicke, Paulien Bunskoek, Kirstin Anderson Hansen, Mette Thybo, Christina Strube, Ursula Siebert

**Affiliations:** 1 Institute for Terrestrial and Aquatic Wildlife Research, University of Veterinary Medicine Hannover, Werftstrasse 6, 25761 Buesum, Germany; anja.reckendorf@tiho-hannover.de (A.R.); kirstin.anderson.hansen@tiho-hannover.de (K.A.H.); 2 Centre for Infection Medicine, Institute for Parasitology, University of Veterinary Medicine Hannover, Buenteweg 17, 30559 Hannover, Germany; christina.strube@tiho-hannover.de; 3 Clinic for Cattle, Working Group Endocrinology, University of Veterinary Medicine Hannover, Bischofsholer Damm 15, 30173 Hannover, Germany; marion.schmicke@landw.uni-halle.de; 4 Dolfinarium, Zuiderzeeboulevard 22, 3841 WB Harderwijk, The Netherlands; paulien.bunskoek@dolfinarium.nl; 5 Fjord & Bælt, Margrethes Pl. 1, 5300 Kerteminde, Denmark; mette@naturama.dk

**Keywords:** harbor porpoise, animal welfare, non-invasive method, exhaled breath, blow sampling, cortisol, stress, diagnostic techniques

## Abstract

**Simple Summary:**

The progress of animal welfare in wildlife conservation and research calls for more non-invasive sampling techniques. In cetaceans, exhaled breath condensate (blow)—a mixture of cells, mucus and fluids expelled through the force of a whale’s exhale—is a unique sampling matrix for hormones, bacteria and genetic material, among others. Especially the detection of steroid hormones, such as cortisol, is being investigated as stress indicators in several species. As the only native cetacean in Germany, harbor porpoises (*Phocoena phocoena*) are of special conservation concern and research interest. So far, strandings and live captures have been the only method to obtain samples from free-ranging individuals, and novel, non-invasive monitoring methods are desirable for this small cetacean species. Hence, three different blow collection devices were tested on harbor porpoises. All samples were analyzed for cortisol using a commercially available immunosorbent assay. The most suitable protocol for sampling, storage and processing is using a sterile 50 mL centrifuge tube. This pilot study shows that cortisol can be detected in the exhale of harbor porpoises, thus paving the way for future studies and most likely successful non-invasive small cetacean health monitoring through blow.

**Abstract:**

Over the last decades, exhaled breath sampling has been established for laboratory analysis in various cetacean species. Due to their small size, the usability of respiratory vapor for hormone assessments was questionable in harbor porpoises (*Phocoena phocoena*). This pilot study compared three different blow collection devices for their suitability in the field and during laboratory processing: a sterile petri dish covered by a Nitex membrane, as well as sterile 50 mL centrifuge tubes with or without manganese(II) chloride as a stabilizer. Collected exhales varied between three, five or ten, depending on feasibility. Hormones were extracted through an ether mix, followed by centrifugal evaporation and cortisol analysis using an immunoassay. Although close to the lower end of the assay’s dynamic range, the ELISA produced results (*n* = 110, 0.102–0.937 ng/mL). Hence, a simple 50 mL centrifuge tube was determined as the best suited blow collection device, while three consecutive exhales proved sufficient to yield results. These findings are promising regarding the suitability of exhaled breath as a matrix for future endocrine and immune system-related studies in harbor porpoises. If further advanced, blow sampling can become an important, non-invasive tool for studying and monitoring health, stress levels and diseases in harbor porpoises.

## 1. Introduction

Marine mammals face a number of growing anthropogenic stressors worldwide, including increasing commercial and recreational activities, chemical and noise pollution, climate change and ecosystem alterations [1,2,3,4]. All of these threats are cumulative stressors, but their collective consequences are often difficult to assess [5,6,7,8]. Stressful and immunocompromising conditions have been shown to cause an increase in infectious disease susceptibility, mortality and reduced reproduction [9,10,11,12]. Cortisol, frequently referred to as the “stress hormone”, is the primary glucocorticoid produced by the mammalian adrenal cortex, secreted in response to stress, and regulating a range of immunological mechanisms of stress adaptation, as well as systems controlling blood pressure and glucose levels [13,14,15]. Usually measured in the blood, cortisol is the main glucocorticoid studied in most (marine) mammals [8,16,17,18]. The typical physiological vertebrate response to a stressor involves a measurable and fast increase in circulating glucocorticoids [19,20,21]. Following an ACTH-dependent diurnal rhythm and seasonal fluctuations, the cortisol level peaks early in the morning and levels off throughout the day [22,23,24,25], making determination of baseline blood concentrations in opportunistically sampled wildlife difficult. Another challenge in wildlife research is that within minutes, the acute stress response associated with pursuit, capture and handling during examinations and sampling procedures is reflected in the blood, potentially obscuring baseline values or long-term stress exposure [26,27,28,29]. Furthermore, not all stress types increase blood cortisol levels [30], and substantial individual cortisol concentration variations are common in different matrices [8,31]. These could be related to intrinsic factors, like the animal’s sex, reproductive stage, body condition and/or age, as well as environmental factors like temperature and season (e.g., [8,24]), and fluctuations in the aforementioned physiological cortisol rhythm that still needs investigation in most cetaceans. Cortisol detection has previously been validated in serum; plasma; tissue; saliva; urine; feces; blubber; keratinous tissues like hair, nails, vibrissae and baleen; as well as respiratory vapor (e.g., [32,33,34,35,36,37,38]). Yet, sample collection from free-ranging marine mammals remains challenging [39].

Throughout the last decade, blow (exhaled breath), as a matrix of organic lung material, has been established as a non-invasive physiological assessment tool for several cetacean species (e.g., humpback whale (*Megaptera novaeangliae*), sperm whale (*Physeter macrocephalus*), long-finned pilot whale (*Globicephala melaena*) and northern bottlenose whale (*Hyperoodon ampullatus*) [40]; and North Atlantic right whale (*Eubalaena glacialis*) [31,41]). Blow sample analysis offers the possibility for immunological, reproductive and adrenal endocrine studies, as well as microbiological and cytological evaluation in several cetacean species, and permits repeated sampling (e.g., [42,43,44,45]). Cetacean lungs have a two layered capillary bed, enabling maximal molecule exchange between circulating blood and air at the pulmonary alveolar membranes [46,47]. Studies in humans additionally revealed that lipophilic molecules like corticosteroids rapidly assimilate into respiratory fluids [48,49]. Due to the exhalation of these respiratory fluids and tissue detritus, the breath matrix contains steroids, cytokines and other biomarkers [50,51,52]. Thus, the hormone status in blow probably reflects the current physiological state or an acute response to a recent stressor [53]. However, blow sampling cannot serve as a real-time stress indicator, since analyses can only be performed in a laboratory.

The harbor porpoise (*Phocoena phocoena*) is widely distributed throughout the northern oceans and is one of the smallest oceanic odontocetes; the average length and weight of an adult is 1.6 m and around 50 kg, respectively [54,55]. Listed as critically endangered on the red list of mammals in Germany and protected under several international agreements (e.g., EU Habitats Directive 92/43/EEC, ASCOBANS, CITES) [56,57], the species has an important status as an ecosystem sentinel and indicator species in the North and Baltic Sea [55,58,59]. Hence, physiology, health and welfare monitoring of free-ranging harbor porpoises, as well as those in rehabilitation and permanent human care, need improvement. A practical, non-invasive stress assessment method is desired as an additional diagnostic and monitoring tool and to increase animal welfare during interventions. The most common method of stress level determination involves blood (e.g., [28,60,61,62]) or fecal sampling, the latter being rarely conducted in harbor porpoises [63]. At present, endocrine blow studies have only been performed on large baleen whales (e.g., [31,64]) and two odontocete species, namely the bottlenose dolphin (*Tursiops truncatus* [38]) and the beluga whale (*Delphinapterus leucas* [43]). Hence, current endocrine monitoring mostly relies on blood collection, while a non-invasive method would provide an obvious animal welfare advance.

Following Burgess’s call for different cetacean species protocols [31], the present analytical pilot study aimed to verify blow as a novel sampling matrix for steroid hormone measurements in harbor porpoises. The study goals were to verify whether hormones can be detected in blow using a commercially available saliva enzyme-linked immunosorbent essay (ELISA), and to determine the most practical collection device for handling in the field and subsequent laboratory analysis.

## 2. Materials and Methods

### 2.1. Blow Sample Collection Devices

#### 2.1.1. Nitex Dish

**Figure 1:**
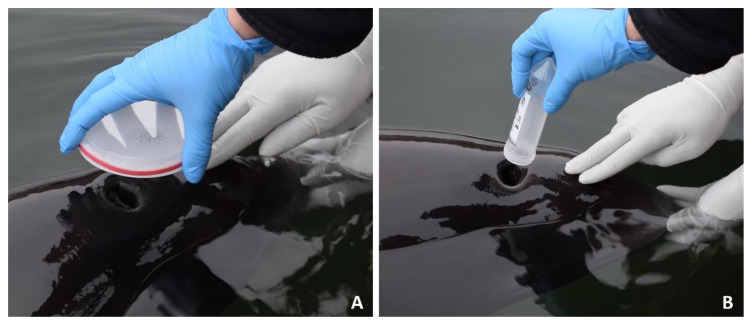
On command, in-water blow sample collection from a trained harbor porpoise. (**A**) Nitex device blow collection, with exhale condensate visible on the petri dish. (**B**) Blow collection into a 50 mL centrifuge tube, visibly fogged up by the exhale.

Following Thompson’s protocol for beluga whale blow sampling [43], polystyrene petri dishes (Item No.: 633181, Greiner Bio-One International GmbH, Kremsmünster, Austria) were covered with nylon mesh of 110 μm pore size (Elko Filtering Co., Miami, FL, USA) fixed with a tight elastic band. Both petri dishes and membranes were additionally wiped down with 70% alcohol to prevent external contamination during assembly. Hereafter, this device will be referred to as “Nitex” (Figure 1A).

#### 2.1.2. Manganese(II) Chloride Tube

During previous health investigations on wild harbor porpoises, researchers of the Institute for Terrestrial and Aquatic Wildlife Research (ITAW), University of Veterinary Medicine Hannover, Germany, had opportunistically collected three or five exhales in sterile 50 mL polypropylene centrifuge tubes (Catalogue No.: 525-0610, VWR International, LLC, Darmstadt, Germany; Figure 1B) as performed in previous studies [38,65,66]. Since these samples had been originally anticipated for sexual hormonal studies, 5 mL of 100 mM manganese(II) chloride (MnCl_2_; Sigma-Aldrich Chemie GmbH, Taufkirchen, Germany) was added in the falcon tubes as stabilizing agent for testosterone [38]. Following this approach, MnCl_2_ was added to 23 newly collected samples. Since previous studies had found no noticeable effect of freezing temperature, delayed freezing, several freeze-thaw cycles or prolonged storage time on the hormone content [67,68,69,70], the older samples (2012–2016, stored at −20 °C) were additionally considered suitable for analysis. This method will hereafter be referred to as “MnCl_2_”.

### 2.2. Study Animals

Samples from both restrained free-ranging individuals and animals in human care were available for this study. To prevent contamination, gloves were worn while handling all equipment and throughout the entire sampling process. All samples were immediately frozen after collection and stored at −20 °C until analysis.

#### 2.2.1. Restrained Free-Ranging Animals

Over the last decade, harbor porpoises that were accidentally caught in Danish pound-nets have undergone health assessments and audiogram recordings performed by the ITAW prior to tagging and subsequent release [71,72] (permits: Danish Ministry of Environment, NST-3446-0016, Danish Ministry of Food, Agriculture and Fisheries, 2010/561-1801). The animals were placed in a floating stretcher for the time of examination, and blow was opportunistically collected when the animal’s habitus and timing allowed it. Three (*n* = 27: 25 MnCl_2_, 2 Falcon) or five (*n* = 4: 1 MnCl_2_, 3 Falcon) consecutive exhales were collected during these opportunistic interventions, depending on the individual’s clinical appearance.

#### 2.2.2. Animals under Human Care

In Europe, harbor porpoises in human care are currently only held under special permits in authorized facilities in Denmark and the Netherlands. For this study, a total of four female porpoises were included. Ten consecutive exhales were collected from these four animals between November 2017 and June 2018. Blow samples were collected non-invasively under constant supervision of an experienced veterinarian during routine clinical examinations or regular training sessions, adhering to ethical principles and respective national and international guidelines for animal experiments. 

The Dolfinarium in Harderwijk, the Netherlands, routinely performs dry-docked health inspections with systematic clinical examinations and sampling on their three females. For the present study, blow was collected onto Nitex dishes during biweekly examinations, which usually take place in the morning.

At Fjord & Bælt in Kerteminde, Denmark, the single animal was trained to routinely exhale on command for medical purposes [27] and was sampled during the regular medical and research training sessions (Figure 1). To prevent enclosure water contamination, the first exhale was not collected. Corresponding Nitex and MnCl_2_ samples were taken in a randomized order at the start and at the end of a training session to compare their suitability for adequate sample collection, storage and subsequent analysis.

### 2.3. Hormone Extraction

#### 2.3.1. Nitex

To extract the entire sample, both the petri dish and the membrane to which the surfactant-rich portion of blow adhered to [69] were processed. The petri dish was rinsed with 5 mL distilled water (H_2_O) to collect any condensate which passed through the mesh onto the dish. Originally, ether mix (30% tert-butylmethylether, AppliChem GmbH, Darmstadt, Germany; 70% petroleum ether, Sigma-Aldrich Chemie GmbH, Taufkirchen, Germany) was used directly on the petri dish. However, since the transparent polystyrene turned blunt on ether contact, this direct method was scrapped in order to prevent potential sample contamination with plastic constituents or additives. For hormone extraction, both H_2_O rinse and membrane were transferred into a sterile 50 mL Falcon tube, and 5 mL ether mix was added. The closed tube was mixed on a shaker (GFL 3006 Analogue Reciprocating Shaker, LAUDA-GFL mbH, Burgwedel, Germany) for 60 min. Following hormone extraction, the membrane was separately centrifuged to collect and include any liquid residue prior to freezing at −80 °C for about 30 min. Due to the large difference in freezing points of H_2_O (0 °C) and ether (around −116.3 °C), the organic ether phase could be easily separated from the frozen H_2_O, followed by evaporation as described below.

#### 2.3.2. MnCl_2_

Hormone extraction in 5 mL ether mix for 60 min could be directly performed within the collection tubes. After hormone extraction, the tubes were frozen at −80 °C for about 30 min to separate the water-solved MnCl_2_ from the ether solution.

Following these extraction steps, the organic phase was transferred into 2 mL microcentrifuge tubes, and the ether was evaporated using an evaporation centrifuge (Concentrator 5301; Eppendorf Vertrieb Deutschland GmbH, Wesseling-Berzdorf, Germany). Prior to sample analysis by a commercial solid phase ELISA, 120 μL zero standard solution for cortisol was added to the remaining invisible hormone pellet.

### 2.4. ELISA

Due to its technical convenience and cost advantage over gas chromatography, a cortisol saliva assay (DEMEDITEC Cortisol free in Saliva ELISA, DES6611, Demeditec Diagnostics GmbH Kiel, Germany) was utilized. This kit is an indirect competitive ELISA including plates coated with a polyclonal rabbit antibody directed against cortisol, enabling determination of free cortisol with high sensitivity. According to the manufacturer’s protocol, the assay’s analytical sensitivity is 0.019 ng/mL, and its dynamic range is between 0.1 and 30 ng/mL. The ELISA cross-reacts with corticosterone (6.2%), cortisone (0.8%), 11-deoxycortisol (2.6%), 11-deoxycortisol (50%), prednisolone (100%), prednisone (0.9%), 7-hydroxyprogesterone (1.3%) and other evaluated steroids (<0.1%). The cross-reactivity of 7-hydroxyprogesterone and other sexual steroids are weak enough to be considered insignificant. All analyses were conducted at room temperature in accordance with the manufacturer’s protocol. First, 50 μL of the cortisol zero standard dissolved hormone samples, two provided controls (low: 0.23–0.47; high: 1.61–3.35 ng/mL), negative controls, as well as cortisol calibrator concentrations at 0, 0.1, 0.4, 1.7, 7 and 30 ng/mL (standards) were thoroughly mixed with 50 μL horseradish peroxidase-conjugated cortisol and incubated for 60 min. Afterwards, the unbound conjugate was decanted, wells were rinsed four times with 300 μL diluted wash solution, and residual droplets were removed by striking the plate on absorbent paper. Subsequently, 200 μL tetramethylbenzidine substrate solution was added to each well and incubated for 30 min in the dark. Color development was stopped by adding 50 μL stop solution to each well, and absorbance was immediately determined at 450 nm in a microplate reader (Infinite^®^ F50, Tecan Trading AG, Männedorf, Switzerland).

Due to the minute sample amount and the pilot nature of the study, the samples were not run in duplicate. Three assays were run to cover all samples. Since glass tubes have been recommended for liquid extraction [41,44,64] and the used materials had not been subjected to petroleum ether as solvent in previous studies, negative controls using 50 µL phosphate buffered saline (PBS; Sigma-Aldrich Chemie GmbH, Taufkirchen, Germany) were run to examine if the long contact time between solvent and collection device materials leads to non-specific binding or produces substances that interfere with the assay. To test extraction efficiency, 50 µL PBS was spiked with 1 ng/mL cortisol standard for mean recovery. Both, zero control and spike-recovery samples were processed as described and run ten times. To determine intra-assay coefficient of variability (CV), one extracted blow sample was reconstituted in 1 mL zero control buffer. In order to have a detectable cortisol concentration in the diluted blow sample, 0.5 ng/mL of cortisol standard was added and the sample run 10 times.

### 2.5. Data Analysis

Raw optical density (OD) values and corresponding cortisol values (ng/mL) automatically calculated using a four-parameter logistic (4PL) curve-fit were generated by the microplate absorbance reader. By using least squares fitting, 4PL modelling generates a predicted standard curve and determines sample concentration, while the R^2^ value represents how well a curve fits the generated data. For proof of correspondence between standard concentrations and sample measurements, the 4PL standard curves of all three conducted assays were visualized.

**Figure 2:**
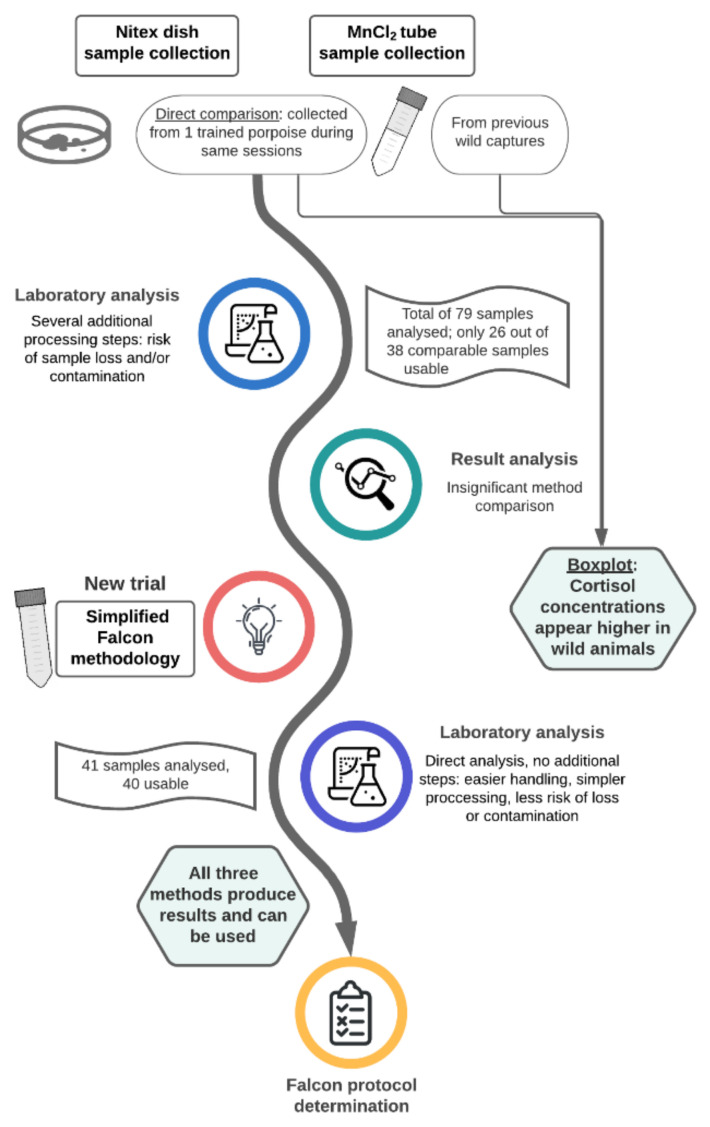
Flowchart clarifying the study’s developing process.

To summarize the data set, simple descriptive statistics (mean, SD) were used. Due to the evolving nature of the study (Figure 2), a direct comparison of all tested methods was technically unfeasible, and boxplots were created to visualize result differences between all three methods employed under similar experimental circumstances. Additionally, MnCl_2_ cortisol levels from a mixed free-ranging, restrained sample pool and one trained harbor porpoise in human care were visualized through boxplots. Shapiro–Wilk’s method was used to test all data for normal distribution, while homogeneity of variance among all sampling methods was verified using the Fligner–Killeen test. All visualizations and calculations were conducted in R (version 4.0.3 [73]), and a *p*-value < 0.05 was considered as significant.

### 2.6. Suggested Sampling Protocol

Sample collection and processing should always be performed wearing clean gloves. Sterile 50 mL centrifuge tubes proved to be the best suited collection device for handling in the field and subsequent laboratory analysis. For successful sample collection, the Falcon tube should be held approximately five to ten cm above the blowhole (Figure 1), while three consecutive exhales are being collected straight into the tube. To avoid excess environmental contamination during blow collection from animals in water, the first exhale should be excluded. The Falcon tube should be closed immediately after sample collection and kept cool until subsequent analysis or stored frozen at −20 °C as soon as possible. For hormone extraction, after adding 5 mL 30% tert-butylmethylether mix to each sample, the tubes should be closed and placed on a shaker for 60 min. Afterwards, the organic phase should be transferred into microcentrifuge tubes, and the ether should be evaporated with an open lid in an evaporation centrifuge. A total of 120 μL zero standard solution for cortisol should be added to the hormone pellet and the manufacturer’s ELISA protocol (Section 2.4.) followed for sample analysis.

## 3. Results

### 3.1. Evolving Sampling Methodology

For direct method comparison, one Nitex and one MnCl_2_ sample were collected in a randomized order from the same individual during 19 sampling sessions. Due to the assay’s dynamic range (0.1–30 ng/mL), two MnCl_2_ and four Nitex samples below 0.1 ng/mL and their counterparts had to be excluded, leaving 13 comparable samples for analysis. However, neither the measured cortisol concentration results for MnCl_2_ (mean = 0.248, SD = 0.123) or Nitex (mean = 0.272, SD = 0.167) nor visualization through boxplot showed a device advantageous for sample collection. As both methods included additional steps during laboratory processing, a more simplistic set-up was sought (Figure 2).

Hence, 50 mL centrifuge tubes without an additional collection membrane or hormone stabilizer, hereafter referred to as “Falcons”, were subsequently trialed (Figure 1B). All Falcon samples were collected from the same individual as the jointly collected Nitex and MnCl_2_ samples.

### 3.2. Sampling Results

**Figure 3:**
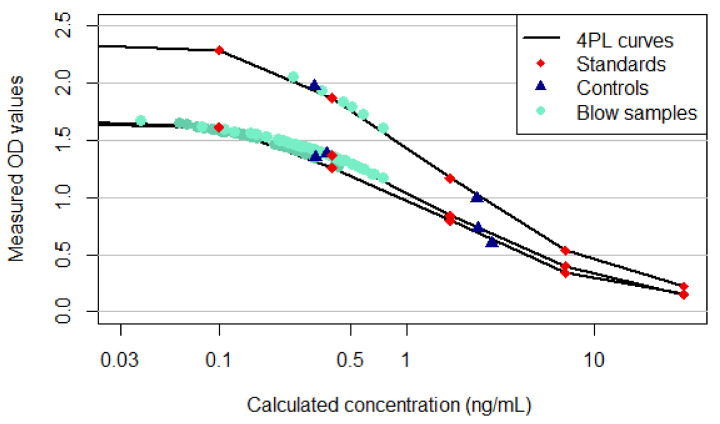
Four-parameter logistic (4PL) curve-fit (calculated concentrations vs. raw optical density (OD) measurements) including 0.1, 0.4, 1.7, 7, 30 ng/mL cortisol standard concentrations (red diamonds), low and high controls (blue triangles) and blow sample data points (turquoise dots) for all ELISA results (*n* = 120). The three different curves correspond to the three assays that were run. As the actual sample concentrations were unknown, 1 was applied as dilution factor for modelling.

All animals revealed a very small, often invisible exhale volume. Hence, measurement and quantification of the actual sample volume were not feasible and standardization proved impossible. Using 4PL-fitted calibration curves by means of the known standard concentrations (0, 0.1, 0.4, 1.7, 7, 30 ng/mL), the unknown sample concentrations were calculated. All measured values defined a corresponding curve (R^2^ = 0.9986/0.9996) and could be included in the study (Figure 3).

For the negative control tests, all measured samples including mean and SD were <0.1 ng/mL, while intra-assay CV was determined to be 12.8%. The mean result of spike-recovery tests was 81.4% (+/−0.12).

**Figure 4:**
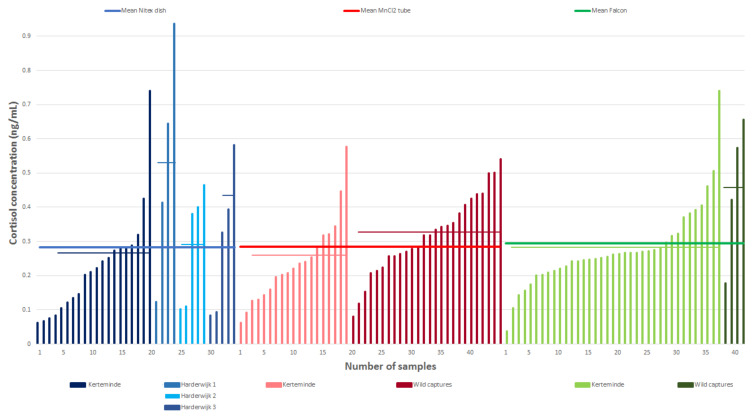
Result distribution among collection devices of all available, but independent samples (*n* = 120). All blue samples were collected with the Nitex dish, all red ones with MnCl_2_ tubes and all green ones with the Falcon method. Means of the different sample types are indicated by a thin line (samples below 0.1 ng/mL were excluded). All wild capture samples derived from different individuals.

For this pilot study, 120 samples (34 Nitex, 45 MnCl_2_ (26 from wild captures), 41 Falcons (4 from wild captures)) were available, which showed a nearly equal mean cortisol concentration between collection devices (Figure 4).

Ten samples, however, showed cortisol values below 0.1 ng/mL (six Nitex, three MnCl_2_, one Falcon) and were hence omitted from further evaluation. The remaining 110 samples consisted of 42 MnCl_2_ samples (0.118–0.577 ng/mL; mean = 0.300), 28 Nitex samples (0.102–0.937 ng/mL; mean = 0.326) and 40 Falcon samples (0.105–0.740 ng/mL; mean = 0.301).

**Figure 5:**
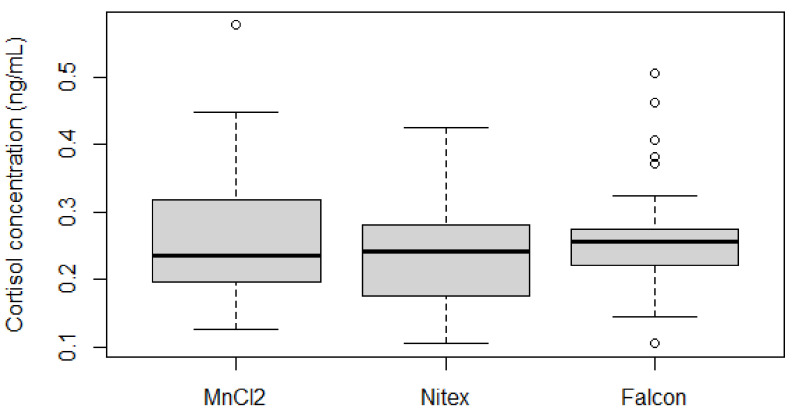
Boxplot comparing the result range and distribution between the three different collection devices among samples from the same individual (*n* = 67). Outliers are shown as circles, while two outliers (0.741 ng/mL Nitex, 0.740 ng/mL Falcon) are not depicted.

A total of 67 samples collected from the same individual under comparable circumstances were used for methodology comparison. A boxplot analysis (Figure 5) showed that the mean cortisol levels of the three methods were similar, while the MnCl_2_ (*n* = 17; mean = 0.259, SD = 0.118) and Nitex (*n* = 16; mean = 0.266, SD = 0.151) results were of greater variability than the Falcon (*n* = 34; mean = 0.280, SD = 0.116) results. However, the variances were not significantly different (Fligner–Killeen: med*X^2^* = 1.209, *p* = 0.546).

**Figure 6:**
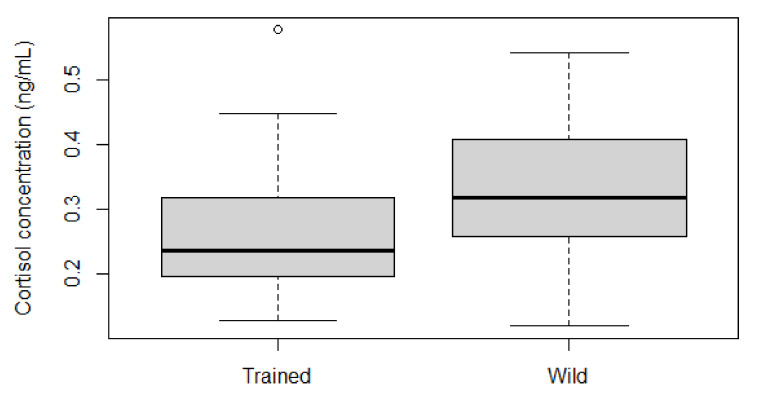
Comparison of MnCl_2_ sample results (*n* = 42) from a trained harbor porpoise (collected prior to and post training) and a free-ranging sample pool, including the samples opportunistically collected between 2012 and 2016. Outliers are depicted as circles.

The 42 MnCl_2_ samples could be further subdivided into 17 samples (mean = 0.259, SD = 0.118) from one trained individual collected under controlled conditions, while 25 samples (mean = 0.327, SD = 0.109) were derived from random live capture events of free-ranging animals. Boxplot comparison (Figure 6) shows that the median of the trained individual lies outside the interquartile range of the free-ranging sample pool, indicating a difference in cortisol concentrations between the two groups, though statistical comparison was not possible due to the different nature (within and between subjects) of the data.

## 4. Discussion

The feasibility of harbor porpoise blow collection and analysis for hormone detection was the primary objective of the present study. Using respiratory vapor for cortisol detection has the demonstrable animal welfare advance of being non-invasive over the currently available options of sampling serum/plasma, tissue and blubber, as well as being easier to obtain than feces, urine or saliva. Although DNA has been previously obtained from harbor porpoise exhalations [66], it was unknown whether hormones could be extracted from their blow. Yet, a non-invasive stress monitoring method for small odontocetes is highly desirable, especially when considering critically endangered small cetaceans. Relating to their aquatic lifestyle with prolonged breath-holding requirements, cetaceans breathe less frequently than terrestrial mammals but exchange large tidal volumes with extreme efficiency during each breath [47,74,75]. While large whales usually produce a visible cloud of respiratory vapor [38,39], the much smaller harbor porpoises take rapid breaths of 2.8–9 L/s exhalation air flow rates [76], with usually invisible exhale condensate. Nonetheless, large whale blow samples produce no visible mucoid deposit and broadly vary in their water and organic content, making sample volume assessments impossible [31,69]. Similarly, we were not able to quantify or standardize collected sample volumes nor account for sample dilution variability. Additionally, the acquired blow amount is highly variable due to extrinsic and intrinsic factors [31], like hormone presence in ambient water being amplified through hormone extraction [45], or exhale intensity determining how much breath condensate can be captured [28,75,77]. Whereas we were able to exclude excessive pool/seawater contamination with high certainty, we still want assay interference through exogenous material and individual differences in exhale strength, both potentially influencing the resulting concentrations, to be the attention of future studies [64].

One clear limitation and challenge of the present study was the limited number of harbor porpoises in human care worldwide. Since all four animals available for the present study were female, sex differences could not be taken into account. However, a discrepancy between factors affecting cortisol levels, like life history traits (sex, age, reproductive state) should be considered in future studies as previously suggested (e.g., [31,78]). Previous studies on larger whales used membranes as the collection device, so the viscous part of the sample could adhere to the mesh [31,36,40,41,43,64,69]. As Nitex membranes showed best sample volume recovery and less assay interference [43], while polystyrene dishes proved most efficient regarding accuracy and precision [64], we chose polystyrene petri dishes covered by Nitex mesh as the main set-up for our trials. Due to the fact that many plastic products release estrogen-like chemicals [79], and endogenous hormones were masked by methodology-related factors in a study that subsequently deemed blow unsuitable for endocrine monitoring in bottlenose dolphins [45], choosing the correct collection and storage materials is crucial for hormonal studies. Since the Falcon method is recommended for further studies and all extractions were performed in Falcons, the negative control tests were performed solely with the 50 mL centrifuge tubes. Their results show that no demonstrable interfering substances were extracted from the used Falcon tubes, while the microcentrifuge tubes used for evaporation have been used for ether extraction without interferences for several years, indicating that the detected values are true cortisol levels from respiratory vapor. Since all available samples were used to demonstrate assay detection, no additional blow samples were available for spike-recovery experiments and PBS was used to mirror diluted blow samples for recovery tests of known hormone doses from Falcons. However, as the recovery was not >90%, it is uncertain if the 50 µL PBS solution accurately reflects blow samples. Therefore, future studies might consider the comparative use of glass tubes for spike recovery tests. Additionally, 1 ng/mL cortisol was spiked into PBS, a concentration much higher than any blow sample measurement. However, as recovery can vary across concentrations, a broader range should be covered in future experiments. Testing for parallelism using serial dilutions was also not possible. Hence, we cannot ascertain that the binding characteristics of blow cortisol are the same as for the calibrator. Not having a full assay validation limits the confidence in the assay’s ability to produce reliable results for its intended use. Nonetheless, now that it was proven that three exhales produce ELISA results, future studies can perform more streamlined tests by collecting several samples at the same time and performing proper validations, including parallelism and spike-recovery tests, as well as correlation with serum values. Several previous studies on cortisol concentrations have used gas or liquid chromatography/mass spectroscopy for sample analysis [32,35,36,38,40], which is more efficient for multiple analytes but also more costly than immunoassay techniques. As the DEMEDITEC Cortisol free in Saliva ELISA has proven reliability in different domestic and wildlife species and matrices, e.g., for successful glucocorticoid determination in koala (*Phascolarctos cinereus*) feces [80], lachrymal fluid and saliva of harbor seals (*Phoca vitulina*) [81], as well as in saliva of different domestic species e.g., [82], this ELISA was chosen for this study. However, any medications containing cortisol, prednisolone or their derivates will significantly influence its results. Hence, respective drug use in study animals needs to be considered as an exclusion criterion for any glucocorticoid ELISA. The presented results show that harbor porpoise blow samples are productive enough to yield cortisol results through ELISA, confirming that immunoassays are a suitable, low-cost analytical method [69]. While Hogg et al. added a broad-spectrum antibiotic to their samples to prevent any degradation during storage [36,38], this was deemed unnecessary in the present study and in other studies [31,41,45,69]. As all older samples from live captures still yielded reasonable and anticipated results, major cortisol degradation over a prolonged storage time (max six years) appeared unreasonable for MnCl_2_ supplemented samples, supporting the longevity and stability of cortisol and the effectiveness of MnCl_2_ as a hormone stabilizer. Still, some level of degradation cannot be excluded. Hence, future studies should investigate the long-term stability (temperature and time) of cortisol and other hormones in Falcon samples without additives.

For improved hormone recovery, the petri dishes required rinsing [31,41,64,69] and centrifugation of the membrane as additional steps, while both Nitex and MnCl_2_ samples needed freezing for solvent separation from additives, making these methods more error-prone during laboratory processing. As the Falcon method required no additional processing steps, it is the superior method from a practical perspective. From an analytical point of view, result comparison between all collection devices also indicates that Falcon samples most likely yielded the most reliable results. The smaller variation in concentration for the Falcon method may indicate assay interference or contamination and sample loss during the additional processing steps of both Nitex and MnCl_2_ methods. This may also be reflected by the fact that six Nitex and three MnCl_2_ samples yielded results <0.1 ng/mL, while only one Falcon sample had to be excluded from the study. Similarly, we cannot exclude some binding of hormone to the dry centrifuge tubes, resulting in the smaller interquartile concentration range of the Falcon samples. Since our original aim was to prove whether blow can be used at all for hormonal analysis, the three devices were not tested with standardized cortisol concentrations. This would, however, be necessary to reliably determine the most dependable collection device and could be tested in a future study. Nevertheless, a significant negative effect through permanent hormone binding to any of the collection devices can probably be excluded, while varying intrinsic factors at different sampling months and time-of-day fluctuations rather than the used sampling method appear reasonable to explain the differences in results.

The anticipated, visibly higher mean cortisol concentrations in free-ranging live captured harbor porpoises in comparison to a trained individual support previous findings that animals performing voluntary husbandry behaviors are less stressed [27], and that individuals probably respond very differently to restraining and interventions, as highly variable cortisol concentrations have been previously observed [28]. Since half of the trained animal’s samples were collected at the end of the training sessions, a potential exercise-induced increase in cortisol levels needs to be considered, which could account for some of the higher values. Furthermore, this would support the conclusion that blow cortisol levels do relate to the actual physiological state of the individual. This indicates that blow is a promising matrix for studying and monitoring acute cortisol levels and probably other hormones.

Prior to this study, some initial blow samples of three opportunistically collected exhales had been analyzed for cortisol, testosterone and estrogen levels by a commercial laboratory, but the majority of these results were below the detection limit. Hence, we anticipated that three exhales were not sufficient to yield results. As Frère et al. collected four to six exhales from bottlenose dolphins for their DNA study [65], a maximum of ten consecutive porpoise exhales was deemed appropriate for a useful endocrine sample and still a feasible amount for one individual. Interestingly, our results show that three exhales are sufficient for successful cortisol detection through immunoassay, if hormone extraction is performed in accordance with the protocol presented here (Section 2.6.). Future studies should investigate whether hormone detection is possible with even fewer exhales, and both saliva and Harderian gland secretion (mucous-like tears) should also be considered as matrices for hormone sampling of animals in human care.

Originally, analyzing both cortisol and dehydroepiandrosterone (DHEA) for cortisol/DHEA ratio comparisons was considered as a possibility, because though cortisol is a major parameter in the endocrine stress response, its use as a single analyte for stress is problematic (e.g., [30,83]). Additionally, the cortisol/DHEA ratio appears to be a better tool for chronic stress evaluation than cortisol alone [82]. However, due to the failure of the initial commercial blow analyses as well as the minimal collected sample amount from harbor porpoises and because even with large whale blow samples most biomarker assays show results close to their detection limit [31], the current analysis was limited to one analyte (cortisol) only. Nevertheless, more sensitive diagnostic techniques or future sample dilution trials may overcome this current limitation and allow for multiple analyses and respective assay accuracy testing. Additionally, microorganisms, DHEA and other parameters may also function as disease biomarkers in the future [82,84,85].

To qualify as a suitable diagnostic tool, hormone measurements from blow must be quantifiable [31]. Examining blow hormone ratios rather than absolute levels has been proposed as a way to account for variable dilutions [41]. Urea as an independent biomarker in large whale blow samples concurred with qualitative respiratory fluid evaluations, suggesting that urea could be used to correct for variable sample dilutions [31]. Hence, if harbor porpoise samples allow for additional assays, we suggest investigating the possibility of also using urea or other internal controls for sample correction in future harbor porpoise blow studies. Additionally, physiological validation should follow as the next step to evaluate blow as an endocrinological monitoring matrix for harbor porpoises. Future studies should compare cortisol levels in blood and blow samples and include environmental water (control) samples to determine the degree of exogenous assay interference and confirm hormone presence within the blow samples rather than it resulting from water contamination. As vertebrate cortisol levels follow a diurnal rhythm and seasonal fluctuations (e.g., [23,86,87]), sampling season and time at collection also need to be considered in future studies. Due to their elusiveness, short surfacing behavior and rapid breathing, blow collection from free-ranging porpoises and other small cetaceans is difficult [54,66,88] and there are still considerable challenges before blow collection can be applied to free-ranging individuals. Yet, this pilot project is the cornerstone of a promising non-invasive health and stress monitoring tool that, if further developed, could improve the overall diagnostic value of blow analysis and be used to assess stress levels, other endocrine functions and diseases in porpoises. It will be particularly useful in rehabilitation where baseline values are not necessarily needed, since individuals can be frequently monitored for trends, and treatments can be amended accordingly. Thus, follow-up future studies could benefit harbor porpoise endocrine research, health management and ultimately conservation. If further advanced, the presented method can additionally be adapted to other small cetacean species in permanent human care and rehabilitation.

## 5. Conclusions

In this pilot study, three different methods were tested for blow sampling from harbor porpoises. We demonstrated that their exhaled breath condensate contains a sufficient amount of organic material to function as a matrix for hormone analysis, so that cortisol can be successfully recovered from all tested devices, and that immunoassays are feasible for hormone detection in harbor porpoise blow. The study identified a sterile 50 mL polypropylene centrifuge tube as being the most suitable device for effectively collecting, storing and analyzing blow samples, while three consecutive exhales produced results. Thus, the method is simple and cost-effective. Future studies on porpoise respiratory samples including validations (physiological validations, determination of reference ranges, etc.), other hormones and internal controls are needed to advance blow sampling as a viable non-invasive technique for assessing the physiological and endocrine condition of porpoises.

## Data Availability

The data presented in this study is contained within the article and is detailed in Figure 3 and Figure 4.
